# The Impact of Optimal Glycemic Control on Tuberculosis Treatment Outcomes in Patients With Diabetes Mellitus: Systematic Review and Meta-Analysis

**DOI:** 10.2196/53948

**Published:** 2024-04-02

**Authors:** Li Zhao, Feng Gao, Chunlan Zheng, Xuezhi Sun

**Affiliations:** 1 Department of Tuberculosis III Wuhan Pulmonary Hospital Wuhan China; 2 Department of Endocrinology Union Hospital, Tongji Medical College Huazhong University of Science and Technology Wuhan China

**Keywords:** optical glycemic control, poor glycemic control, tuberculosis treatment, diabetes mellitus, health management, healthcare, health care, glycemic control, tuberculosis, TB, DM, diabetes, systematic review, meta-analysis, risks, treatment, treatment outcome, mortality, patients, burden, disease burden

## Abstract

**Background:**

Diabetes mellitus (DM) increases the risk of developing tuberculosis (TB), and optimal glycemic control has been shown to reduce the risk of complications and improve the TB treatment outcomes in patients with DM.

**Objective:**

This study aims to investigate the role of glycemic control in improving TB treatment outcomes among patients with DM.

**Methods:**

MEDLINE, Embase, and the Cochrane Central Register of Controlled Trials databases were searched for randomized controlled trials (RCTs) assessing the impact of oral glycemic control in patients with TB who have DM. Outcomes of interest were radiological findings, treatment success, sputum positivity, and mortality. Evaluations were reported as risk ratios (RRs) with 95% CIs using weighted random-effects models.

**Results:**

The analysis included 6919 patients from 7 observational studies. Our meta-analysis showed significant differences between patients with optimal glycemic control and those with poor glycemic control with regard to improved treatment outcomes (RR 1.13, 95% CI 1.02-1.25; *P*=.02; *I*²=65%), reduced sputum positivity (RR 0.23, 95% CI 0.09-0.61; *P*=.003; *I*²=66%), and fewer cavitary lesions (RR 0.59, 95% CI 0.51-0.68; *P*<.001; *I*²=0%) in radiological findings. There was no significant difference between the 2 groups in terms of mortality (RR 0.57, 95% CI 0.22-1.49; *P*=.25; *I*²=0%), multilobar involvement (RR 0.57, 95% CI 0.22-1.49; *P*=.25; *I*²=0%) on radiologic examination, and upper lobe (RR 0.94, 95% CI 0.76-1.17; *P*=.58; *I*²=0%) and lower lobe (RR 1.05, 95% CI 0.48-2.30; *P*=.91; *I*²=75%) involvement on radiologic examination.

**Conclusions:**

We concluded that optimal glycemic control is crucial for reducing susceptibility, minimizing complications, and improving treatment outcomes in patients with TB with DM. Emphasizing effective health management and health care strategies are essential in achieving this control. Integrating comprehensive care among patients with TB with DM will enhance patient outcomes and alleviate the burden of disease in this population.

**Trial Registration:**

PROSPERO CRD42023427362; https://www.crd.york.ac.uk/prospero/display_record.php?RecordID=427362

## Introduction

Tuberculosis (TB) poses an escalating public health threat, particularly in lower- and middle-income countries [1]. The World Health Organization estimates that approximately one-fourth of the world's population has been infected with TB-causing bacteria [2], with 10.6 million individuals diagnosed with TB in 2021, leading to 1.6 million deaths [1]. Risk factors for TB are divided into 2 main categories, that is, people recently infected with TB and those with an immunocompromised status, including those with HIV, diabetes mellitus (DM), transplants, malnourishment, and tobacco use, and those receiving immunosuppressants [1,2].

DM is also a growing concern, increasing the likelihood of several infections and complications [3]. With 425 million individuals affected in 2017 and an estimated 629 million expected to be impacted by 2045, DM increases the risk of TB incidence by 2-4 folds. Furthermore, it is associated with poor outcomes, doubling the risk of mortality during treatment [4,5]. In the long term, hyperglycemia and poor glycemic control (PGC) impair immunity, leading to immunosuppression and increased susceptibility to TB [1].

Poor treatment outcomes have been associated with patients with TB, including treatment failure, recurrence, delayed culture conversion, and death [6]. Optimal glycemic control (OGC) has been shown to improve TB outcomes by enhancing phagocytic activity and other immunological defense mechanisms [6]. Nevertheless, some studies have found no significant improvement in TB treatment outcomes through glycemic control [7]. As a result, there is a need to examine the current data to establish the relationship between the 2 factors. This paper aims to review the current literature and reach a conclusion regarding the impact of OGC on TB treatment outcomes in patients with DM.

## Methods

### Overview

The PRISMA (Preferred Reporting Items for Systematic Reviews and Meta-Analyses) guidelines and the risk of bias assessed using AMSTAR (A Measurement Tool to Assess systematic Reviews) 2 were both used when performing this meta-analysis [8,9]. This study is registered on PROSPERO (The International Prospective Register of Systematic Reviews; ID CRD42023427362).

### Ethical Considerations

Since the information was accessible to the general public, institutional review board approval was not necessary.

### Data Sources and Search Strategy

MEDLINE, Embase, and the Cochrane Central Register of Controlled Trials were comprehensively searched from inception through May 2023 by 2 independent reviewers (LZ and XS). We extracted studies based on abstracts and titles. A full-text appraisal was sought when required. MeSH (Medical Subject Headings) phrases and keywords were used to formulate search strategies (Table 1).

**Table 1:** Search strategy used in each database.

Database (articles retrieved)	Search strategy
MEDLINE (146 results)	(“Tuberculosis”[MeSH] OR “Tuberculosis” OR “TB”) AND (“Diabetes Mellitus”[MeSH] OR “Diabetes Mellitus” OR “DM”) AND (“Glycemic Control”[MeSH] OR “Glycemic Control” OR “Blood Glucose Control” OR “Blood Sugar Control”) AND (“Randomized Controlled Trial”[Publication Type] OR “Clinical Trial”[Publication Type] OR “Observational Study”[Publication Type])
Embase (56 results)	(“tuberculosis”/exp OR “tuberculosis” OR “TB”) AND (“diabetes mellitus”/exp OR “diabetes mellitus” OR “DM”) AND (“glycemic control”/exp OR “glycemic control” OR “blood glucose control” OR “blood sugar control”) AND (“randomized controlled trial”/exp OR “clinical trial”/exp OR “observational study”/exp)
Cochrane Central Register of Controlled Trials (25 results)	(“Tuberculosis” OR “TB”) AND (“Diabetes Mellitus” OR “DM”) AND (“Glycemic Control” OR “Blood Glucose Control” OR “Blood Sugar Control”) AND (“Randomized Controlled Trial” OR “Clinical Trial” OR “Observational Study”)

### Study Selection

We included studies if they (1) were randomized controlled trials (RCTs) or analyses of RCTs that determined the impact of OGC on treatment outcomes of TB in patients with DM in different interventional arms; (2) reported radiological findings including cavitary lesions, multilobar involvement, and upper and lower lobe involvement; treatment success; sputum positivity; or mortality; or (3) included patients with a diagnosis of TB and DM. We also included observational studies that reported the aforementioned radiological findings, treatment success, sputum positivity, and mortality. A third investigator (XS) was consulted in case of any disagreement regarding study selection. All articles were then uploaded to EndNote Reference Library (version X7.5; Clarivate Analytics) software to remove any duplicates.

### Data Extraction and Assessment of Study Quality

Two reviewers (FG and CZ) independently extracted from the selected studies the characteristics of the studies, patient demographics, summary events, number of events, sample sizes, and treatment type. The quality of the included studies was assessed using the Newcastle-Ottawa Scale across 6 key domains: selection bias, performance bias, detection bias, attrition bias, reporting bias, and other bias. This systematic evaluation aimed to enhance the reliability of our findings by critically appraising the internal validity of each study. To enhance the reliability of our quality assessments, 2 independent reviewers (FG and CZ) conducted the evaluations. In instances of disagreement, a third investigator (XS) was consulted, and consensus was reached through discussion. The process of quality assessment was conducted systematically and transparently, ensuring a rigorous evaluation of each study's methodological robustness.

### Statistical Analysis

Radiological findings, consisting of cavitary lesions, multilobar involvement, upper lobe involvement, and lower lobe involvement, were one of the outcomes of interest. Other outcomes were treatment success, sputum positivity, and mortality. RevMan (version 5.4.1; The Cochrane Collaboration) was used to conduct the meta-analysis. The outcomes of interest were provided as risk ratios (RRs), assessing the association between exposure and disease, indicating the risk of developing the disease in the exposed group versus the nonexposed group with 95% CIs and aggregated using an inverse variance–weighted random effects model. Forest plots were used to graphically display the pooled analyses. The Higgins *I*^2^ was used to assess heterogeneity between trials; a value of 25%-50% was regarded as low, 50%-75% as moderate, and >75% as serious. In all cases, a *P* value less than .05 was considered significant.

## Results

### Search Results

**Figure 1:**
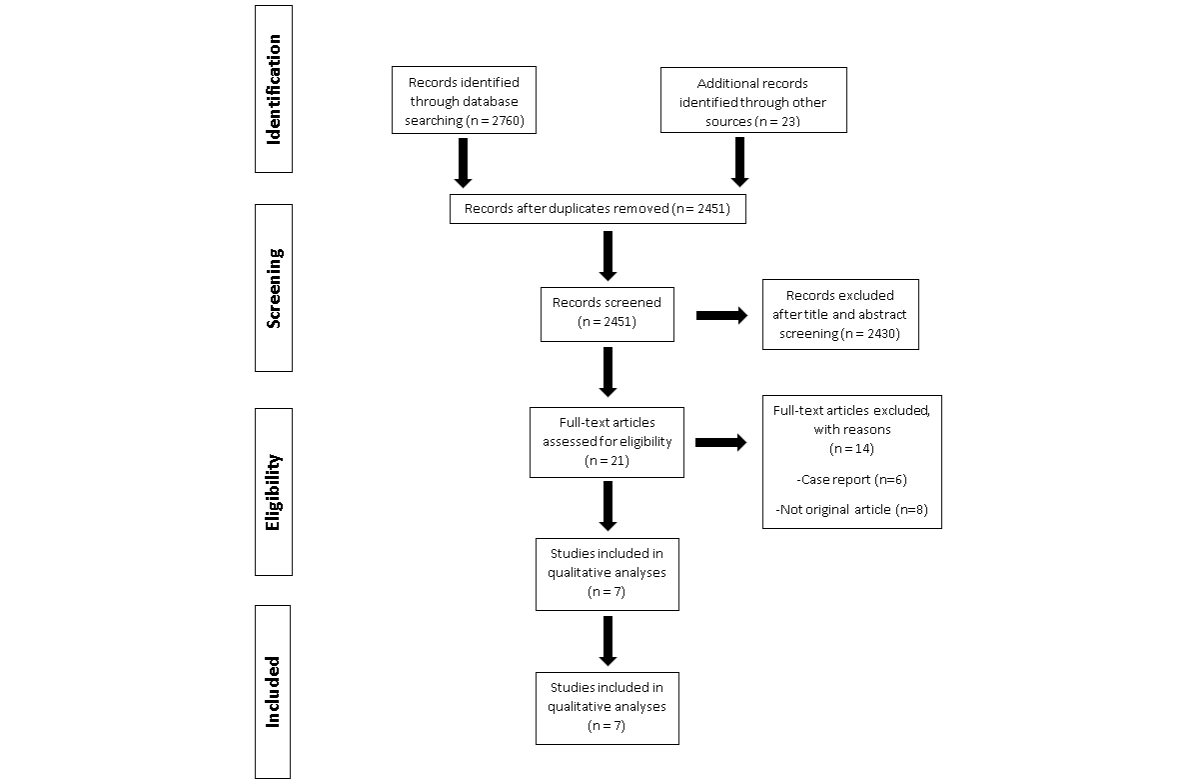
PRISMA (Preferred Reporting Items for Systematic Reviews and Meta-Analyses) flow diagram of study identification for the meta-analysis.

Our initial search yielded 2760 potentially relevant articles, of which 21 were selected for full-text review. Upon further exclusions, 7 observational studies, with a total of 6919 patients, were shortlisted for data extraction [10-16]. The PRISMA flowchart in Figure 1 shows the literature search in detail.

### Study Characteristics and Quality Assessment

Study characteristics and baseline demographics are summarized in Table 2.

Observational studies were assessed to be of moderate to high quality, achieving scores from 4 to 6 out of a maximum of 9 on the Newcastle-Ottawa scale (Table 3).

**Table 2:** Baseline characteristics of the included studies.

Study (year)	Sites	Design	Intervention and exposure	Study duration	Participants
Chiang et al [10] (2015)	North, south, and east Taiwan	Retrospective cohort study	Criteria for classification of glycemic control at baseline were as follows: HbA_1c_^a^<7—glycemic control arm; HbA_1c_ of 7-9—glycemic control–less stringent arm; and HbA_1c_>9—poor glycemic control arm.	2005-2010	705 patients with DM^b^ with culture-positive pulmonary TB^c^ of both sexes; age not reported irrespective of HIV status; diagnosed with DM within 3 months of initiation of ATT^d^; included 768 patients with TB without DM.
Mi et al [11] (2013)	South China	Retrospective cohort study	Criteria for classification of glycemic control (baseline, 2 months, and 6 months): good glycemic control—FBG^e^<7.0 mmol/L; poor glycemic control—FBG 7.0-10 mmol/L; and bad glycemic control—FBG>10.0 mmol/L.	2011-2012	189 patients with pulmonary or extrapulmonary TB with DM of all age groups, both sexes, and HIV status not reported; included 1400 patients with TB without DM.
Magee et al [12] (2013)	Lima, Peru	Retrospective/prospective cohort study	Based on documentation of control in records, an FBG level below median, or an FBG level of <136 mmol/L. Exact criteria are not specified. Details on glucose lowering treatment are available: OHA^f^ only—56 participants; insulin only—16 participants; both—26 participants.	2005-2008	Selected group of patients with TB screened for high risk of developing multidrug-resistant TB (people with presumptive multidrug-resistant TB); 1485 patients with TB without DM and 186 patients with TB and DM receiving new treatment or retreatment, aged ≥15 years, of either sex, regardless of HIV status were also included.
Nandakumar et al [13] (2013)	Kerala, India	Retrospective cohort study	Criteria for glycemic control: assessed 3 times, at least 1 month apart, and at least in 1 control program. Those fulfilling all 3 criteria were classified as having a “known” diabetic control status. Those with all of the following 3 values less than the cutoff were classified as “controlled”: FBS level of <100 mg, postprandial blood sugar or random blood sugar level of <140 mg.	2010-2011	667 patients with TB and DM, new or retreatment, pulmonary or extrapulmonary TB, aged ≥15 years, belonging to either sex, irrespective of HIV status.
Park et al [14] (2012)	South Korea	Retrospective cohort study	Criteria for glycemic control assessment at baseline were as follows: glycemic control— HbA_1c_<7; poor glycemic control—HbA_1c_≥7.	2005-2009	New patients with pulmonary TB, 96 with TB and DM, and 148 with TB without DM, aged ≥18 years, of either sex, whose HIV status is not reported.
Yoon et al [15] (2017)	South Korea	Prospective cohort study	Criteria for classifying glycemic control at baseline were as follows: glycemic control—HbA_1c_<7; less stringent glycemic control—HbA_1c_ level of 7-8.99; poor glycemic control—HbA_1c_≥9.	2012-2014	New patients with pulmonary TB: 157 with TB and DM and 504 with TB without DM, aged 18-75 years, of either sex, excluding those with an HIV-positive status.
Mahishale et al [16] (2017)	India	Prospective cohort study	Glycemic control was defined at baseline: poor glycemic control—HbA_1c_≥7%; optimal glycemic control—HbA_1c_<7%; no mention of NGSP^g^ certification and standardized to the DCCT^h^ assay.	2012-2014	675 new patients with pulmonary TB belonging to either sex, age group unspecified, and excluding known HIV-positive cases.

^a^HBA_1c_: hemoglobin A_1c_.

^b^DM: diabetes mellitus.

^c^TB: tuberculosis.

^d^ATT: anti-tuberculosis treatment.

^e^FBG: fasting blood glucose.

^f^OHA: oral hypoglycemic agent.

^g^NGSP: National Glycohemoglobin Standardization Program.

^h^DCCT: Diabetes Control and Complications Trial.

**Table 3:** Quality assessment of included observational studies using the Newcastle-Ottawa Scale.

Study (year)	Selection					Comparability		Outcome				Total, n
	S1	S2	S3	S4	C		O1		O2	O3		
Chiang et al [10] (2015)		✓		✓	✓					✓	4	
Mi et al [11] (2013)	✓	✓	✓	✓	✓					✓	6	
Magee et al [12] (2013)			✓				✓		✓		3	
Nandakumar et al [13] (2013)	✓	✓		✓			✓			✓	5	
Park et al [14] (2012)	✓	✓			✓		✓			✓	5	
Yoon et al [15] (2017)			✓	✓	✓					✓	4	
Mahishale et al [16] (2017)	✓		✓	✓	✓					✓	5	

### Outcomes

#### Treatment Outcome

**Figure 2:**
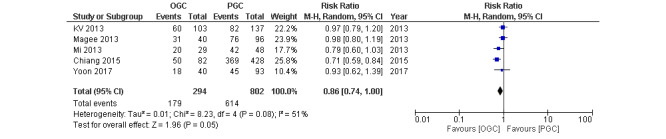
Forest plot comparing treatment outcomes among patients with optimal glycemic control (OGC) and those with poor glycemic control (PGC) [10-13,15].

Five studies reported treatment outcomes among patients with TB, which included patients who completed the treatment and were completely cured. Our meta-analysis revealed a significant difference in treatment outcomes between patients with OGC and those with PGC (RR 0.86, 95% CI 0.74-1.00; *P*=.05; *I*²=51%; Figure 2).

#### Sputum Positivity Following Treatment

**Figure 3:**
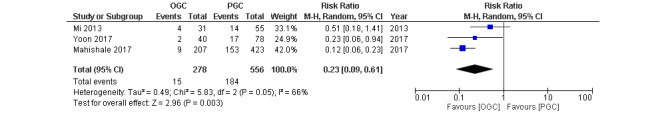
Forest plot comparing sputum positivity following treatment between patients with optimal glycemic control (OGC) and those with poor glycemic control (PGC) [11,15,16].

Three studies reported sputum positivity as an outcome among patients with TB. Sputum positivity was more likely among patients with PGC than among those with OGC (RR 0.23, 95% CI 0.09-0.61; *P*=.003; *I*²=66%; Figure 3).

#### Mortality

**Figure 4:**
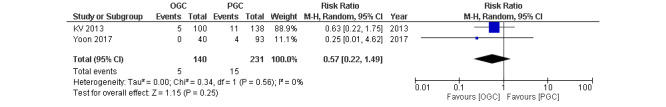
Forest plot comparing mortality between patients with optimal glycemic control (OGC) and those with poor glycemic control (PGC) [13,15].

Two studies reported mortality as an outcome among patients with TB. Mortality was not significantly different between patients with OGC and those with PGC (RR 0.57, 95% CI 0.22-1.49; *P*=.25; *I*²=0%; Figure 4).

#### Radiological Outcomes

A total of 3 studies reported radiological findings among patients with TB. These findings were further divided into cavitary lesions, multilobar involvement, isolated upper lobe involvement, and lower lobe involvement.

#### Cavitary Lesions

**Figure 5:**
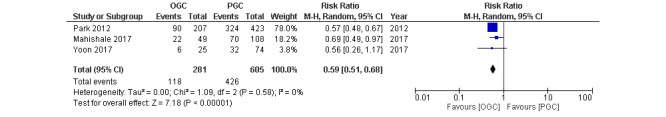
Forest plot comparing cavitary lesions as a radiological outcome between patients with optimal glycemic control (OGC) and those with poor glycemic control (PGC) [14-16].

Three studies reported cavitary lesions as one of their radiological findings. Patients with OGC had a lower risk of cavitary lesions than those with PGC (RR 0.59, 95% CI 0.51-0.68; *P*<.001; *I*²=0%; Figure 5).

#### Multilobar Involvement

**Figure 6:**
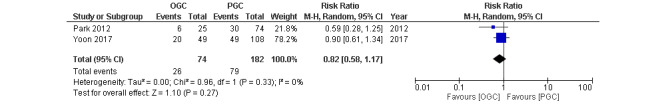
Forest plot comparing multilobar involvement as a radiological outcome between patients with optimal glycemic control (OGC) and those with poor glycemic control (PGC) [14,15].

Among the 3 studies reporting radiological findings, 2 reported multilobar involvement. There was no significant difference between patients with OGC and those with PGC (RR 0.82, 95% CI 0.58-1.17; *P*=.27; *I*²=0%; Figure 6).

#### Isolated Upper or Lower Lobe Involvement

**Figure 7:**
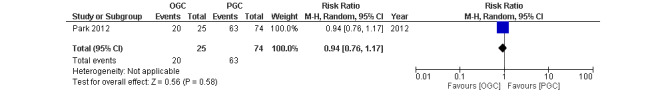
Forest plot comparing isolated upper lobe involvement as a radiological outcome between patients with optimal glycemic control (OGC) and those with poor glycemic control (PGC) [14].

**Figure 8:**
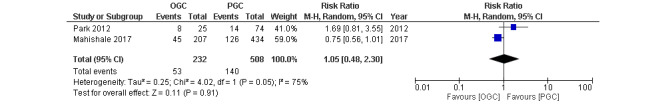
Forest plot comparing isolated lower lobe involvement as a radiological outcome between patients with optimal glycemic control (OGC) and those with poor glycemic control (PGC) [14,16].

Two studies reported isolated lower lobe involvement, while 1 study reported isolated upper lobe involvement as their radiological outcomes. There was no significant difference between patients with OGC and those with PGC for both upper (RR 0.94, 95% CI 0.76-1.17; *P*=.58; *I*²=0%) and lower lobe involvement (RR 1.05, 95% CI 0.48-2.30; *P*=.91; *I*²=75%; Figures 7 and 8).

## Discussion

### Principal Findings

This meta-analysis evaluating the impact of OGC among patients with TB reports several key findings. First, patients with OGC demonstrated a decreased risk of the aforementioned treatment outcomes. Second, we observed decreased sputum positivity in patients with OGC compared to that in patients with suboptimal glycemic control. Third, there was a decreased risk of cavitary lesions on radiologic examination among patients with OGC.

The analysis revealed a significant improvement in treatment outcomes among patients with OGC compared to those with PGC. This finding is consistent with those of previous studies highlighting the importance of glycemic control in reducing the risk of complications and improving treatment responses in individuals with DM [17,18]. The ability to achieve OGC may enhance the body's immune response, leading to better control of the TB infection and a more favorable treatment outcome [19]. Moreover, our analysis showed that patients with OGC had decreased sputum positivity. This may be attributable to the quicker clearance of bacteria from the airways, resulting in earlier detection. The decreased sputum positivity following OGC points toward the clinical benefits and importance of achieving OGC among patients with TB.

There was no significant difference in mortality between patients with OGC and those with PGC. This result contradicts the findings of previous studies that have linked DM to an increased risk of mortality among patients with TB [20]. It is important to note that the studies included in the meta-analysis might have varied in terms of follow-up duration and other factors that could influence mortality outcomes. Further research is needed to better understand the relationship among glycemic control, TB treatment outcomes, and mortality.

Patients with OGC had a lower risk of cavitary lesions than those with PGC, according to our analysis. Cavitary lesions are indicative of advanced disease and are associated with an increased risk of TB transmission [21]. This finding suggests that OGC may aid in preventing the progression of disease and decreasing the risk of transmission. However, multilobar involvement, isolated upper lobe involvement, and isolated lower lobe involvement did not differ significantly between patients with OGC and those with PGC. This indicates that OGC may have a limited effect on the distribution of TB lesions in the lungs [22].

Publication bias—the tendency of studies with positive or statistically significant results to be published more readily than those with null or negative results—is a concern in meta-analyses. However, due to the limited number of studies available for each outcome, the statistical power of Egger and Begg tests may have been compromised. As such, the ability to draw definitive conclusions regarding publication bias is constrained. To mitigate this limitation, we attempted to include a broad range of studies by searching multiple databases and imposing minimal restrictions on study design. Additionally, we actively sought unpublished studies, conference abstracts, and gray literature to reduce the impact of publication bias. However, despite these efforts, it is essential to interpret our findings with caution, considering the potential influence of publication bias on the reported results.

Overall, our findings indicate that OGC is essential for enhancing TB treatment outcomes and lowering the risk of advanced disease. In patients with TB, health care providers should consider screening for DM and managing glycemic control [23]. To thoroughly comprehend the relationship among DM, glycemic control, and TB outcomes, additional research is required.

In evaluating the robustness of our findings, we conducted a thorough quality assessment using the Cochrane Risk of Bias Tool, systematically appraising studies across key domains. While our inclusive approach aimed to minimize publication bias by considering a broad range of studies and actively seeking unpublished data, the limitations in conclusively identifying and mitigating publication bias should be acknowledged. Regarding heterogeneity, variations in study design, patient populations, glycemic control thresholds, and outcome measurements were identified as potential sources. These factors introduce complexity and may limit the generalizability of our results. Clinicians should interpret our findings with caution, considering the diverse contexts and populations represented in the included studies. Future research addressing standardized definitions of glycemic control and consistent outcome measures will contribute to a more nuanced understanding of the relationship between glycemic control and TB treatment outcomes.

Our comprehensive findings have clinical implications for both individual patient care and public health strategies in the context of TB management. Notably, OGC not only improves overall TB treatment outcomes but also has emerged as a critical factor in reducing its infectiousness, as evidenced by the observed decreases in sputum positivity and the lower risk of cavitary lesions. The reduction in sputum positivity implies a potential limitation on TB transmission, presenting a dual benefit for both individual patients’ well-being and broader public health goals. Additionally, the lower risk of cavitary lesions, indicative of advanced TB disease, signifies a potential avenue for mitigating the contagiousness of patients with TB. Clinicians should emphasize the importance of achieving and sustaining OGC, recognizing its dual impact on individual health and community-level TB transmission. Integrated health care strategies focusing on glycemic control in patients with TB are vital, providing actionable insights for clinicians and public health practitioners alike and contributing to the overarching goal of TB control and prevention. While our findings provide valuable insights into the association between glycemic control and TB outcomes in patients with DM, generalizing these results to a wider population requires caution. The unique characteristics of patients with TB with DM, the potential variations in glycemic control thresholds, and the diverse health care settings may limit the direct applicability of our findings to those without DM or with different comorbidities. Furthermore, the prevalence of observational studies in our analysis introduces biases that may affect the external validity of our results. Caution is advised in extending these findings to diverse patient populations, and future research should explore the relationship between glycemic control and TB outcomes in broader contexts, considering various comorbidities and health care settings

However, there are some limitations to consider in interpreting the results. First, the included studies were observational in nature, which may introduce biases, such as selection bias and confounding factors that could influence the results. Moreover, an observational study design has variability in its population, selective and incomplete reporting, and improper randomization, which may contribute to the heterogeneity observed in this study. Second, heterogeneity was observed in some of the studies, which may be attributed to differences in study design, patient populations, and glycemic control thresholds across the included studies. The limited research carried out on this topic shows that OGC is an important predictor of outcomes in patients with TB; however, there are some discrepancies, which may raise doubt among clinicians. Thus, future investigation on this topic is warranted in order to derive a robust conclusion.

### Conclusions

In conclusion, our meta-analysis compiled data from observational studies assessing glycemic control in patients with TB, and our results suggest that OGC may have a significant impact on improving treatment outcomes and reducing sputum positivity in patients with TB. However, no significant difference was found in mortality between patients with OGC and those with PGC. OGC was also associated with a lower risk of cavitary lesions but had no significant effect on multilobar or isolated upper or lower lobe involvement. These findings highlight the importance of early detection of TB in patients with DM so that OGC can be provided promptly to those patients, thus improving their outcomes. This topic warrants further research, especially RCTs focusing on mortality and other outcomes in different severities of TB among patients with DM.

## Appendix

Multimedia Appendix 1 PRISMA checklist.

## Abbreviations

AMSTAR: A Measurement Tool to Assess systematic Reviews
DM: diabetes mellitus
MeSH: Medical Subject Headings
OGC: optimal glycemic control
PGC: poor glycemic control
PRISMA: Preferred Reporting Items for Systematic Reviews and Meta-Analyses
PROSPERO: The International Prospective Register of Systematic Reviews
RCT: randomized controlled trial
RR: risk ratio
TB: tuberculosis

## References

[1] Kansal HM, Srivastava S, Bhargava SK (1942). Diabetes mellitus and tuberculosis. J Int Med Sci Acad.

[2] (2023). Tuberculosis. World Health Organization.

[3] Casqueiro J, Casqueiro J, Alves C (2012). Infections in patients with diabetes mellitus: a review of pathogenesis. Indian J Endocrinol Metab.

[4] van Crevel R, Critchley JA (2021). The interaction of diabetes and tuberculosis: translating research to policy and practice. Trop Med Infect Dis.

[5] Al-Rifai RH, Pearson F, Critchley JA, Abu-Raddad LJ (2017). Association between diabetes mellitus and active tuberculosis: a systematic review and meta-analysis. PLoS One.

[6] Jørgensen Marit Eika, Faurholt-Jepsen D (2014). Is there an effect of glucose lowering treatment on incidence and prognosis of tuberculosis? A systematic review. Curr Diab Rep.

[7] Shewade HD, Jeyashree K, Mahajan P, Shah AN, Kirubakaran R, Rao R, Kumar AMV (2017). Effect of glycemic control and type of diabetes treatment on unsuccessful TB treatment outcomes among people with TB-Diabetes: A systematic review. PLoS One.

[8] Liberati A, Altman DG, Tetzlaff J, Mulrow C, Gøtzsche Peter C, Ioannidis JPA, Clarke M, Devereaux PJ, Kleijnen J, Moher D (2009). The PRISMA statement for reporting systematic reviews and meta-analyses of studies that evaluate healthcare interventions: explanation and elaboration. BMJ.

[9] Shea BJ, Reeves BC, Wells G, Thuku M, Hamel C, Moran J, Moher D, Tugwell P, Welch V, Kristjansson E, Henry DA (2017). AMSTAR 2: a critical appraisal tool for systematic reviews that include randomised or non-randomised studies of healthcare interventions, or both. BMJ.

[10] Chiang CY, Bai KJ, Lin HH, Chien ST, Lee JJ, Enarson DA, Lee T, Yu M (2015). The influence of diabetes, glycemic control, and diabetes-related comorbidities on pulmonary tuberculosis. PLoS One.

[11] Mi F, Tan S, Liang L, Harries AD, Hinderaker SG, Lin Y, Yue W, Chen X, Liang B, Gong F, Du J (2013). Diabetes mellitus and tuberculosis: pattern of tuberculosis, two‐month smear conversion and treatment outcomes in uangzhou, hina. Tropical Med Int Health.

[12] Magee M, Bloss E, Shin S, Contreras C, Huaman HA, Ticona JC, Bayona J, Bonilla C, Yagui M, Jave O, Cegielski J (2013). Clinical characteristics, drug resistance, and treatment outcomes among tuberculosis patients with diabetes in Peru. Int J Infect Dis.

[13] Nandakumar KV, Duraisamy K, Balakrishnan Shibu, Sagili Karuna D, Satyanarayana Srinath, Enarson Donald A (2013). Outcome of tuberculosis treatment in patients with diabetes mellitus treated in the revised national tuberculosis control programme in Malappuram District, Kerala, India. PLoS One.

[14] Park SW, Shin JW, Kim JY, Park IW, Choi BW, Choi JC, Kim YS (2012). The effect of diabetic control status on the clinical features of pulmonary tuberculosis. Eur J Clin Microbiol Infect Dis.

[15] Yoon YS, Jung J, Jeon EJ, Seo H, Ryu YJ, Yim J, Kim YH, Lee B, Park YB, Lee BJ, Kang H, Choi JC (2017). The effect of diabetes control status on treatment response in pulmonary tuberculosis: a prospective study. Thorax.

[16] Mahishale Vinay, Avuthu Sindhuri, Patil Bhagyashri, Lolly Mitchelle, Eti Ajith, Khan Sujeer (2017). Effect of poor glycemic control in newly diagnosed patients with smear-positive pulmonary tuberculosis and type-2 diabetes mellitus. Iran J Med Sci.

[17] Rodríguez-Gutiérrez R, Montori VM (2016). Glycemic control for patients with type 2 diabetes mellitus: our evolving faith in the face of evidence. Circ: Cardiovascular Quality and Outcomes.

[18] Bitew ZW, Alemu A, Jember DA, Tadesse E, Getaneh FB, Sied Awole, Weldeyonnes M (2023). Prevalence of glycemic control and factors associated with poor glycemic control: a systematic review and meta-analysis. Inquiry.

[19] Ngo MD, Bartlett S, Ronacher K (2021). Diabetes-associated susceptibility to tuberculosis: contribution of hyperglycemia vs. dyslipidemia. Microorganisms.

[20] Gautam S, Shrestha N, Mahato S, Nguyen TPA, Mishra SR, Berg-Beckhoff G (2021). Diabetes among tuberculosis patients and its impact on tuberculosis treatment in South Asia: a systematic review and meta-analysis. Sci Rep.

[21] Urbanowski ME, Ordonez AA, Ruiz-Bedoya CA, Jain SK, Bishai WR (2020). Cavitary tuberculosis: the gateway of disease transmission. Lancet Infect Dis.

[22] Mahishale V, Avuthu Sindhuri, Patil Bhagyashri, Lolly Mitchelle, Eti Ajith, Khan Sujeer (2017). Effect of poor glycemic control in newly diagnosed patients with smear-positive pulmonary tuberculosis and type-2 diabetes mellitus. Iran J Med Sci.

[23] Gurukartick J, Murali L, Shewade HD, Jacob AG, Samy MM, Dheenadayal D, Aslesh OP, Marimuthu G, Ananthakrishnan R, Krishnan N (2019). Glycemic control monitoring in patients with tuberculosis and diabetes: a descriptive study from programmatic setting in Tamil Nadu, India. F1000Res.

